# Effect of Baloxavir and Oseltamivir in Combination on Infection with Influenza Viruses with PA/I38T or PA/E23K Substitutions in the Ferret Model

**DOI:** 10.1128/mbio.01056-22

**Published:** 2022-08-08

**Authors:** Paulina Koszalka, Ankita George, Vijaykrishna Dhanasekaran, Aeron C. Hurt, Kanta Subbarao

**Affiliations:** a WHO Collaborating Centre for Reference and Research on Influenza at The Peter Doherty Institute for Infection and Immunity, Melbourne, Victoria, Australia; b Biomedicine Discovery Institute and Department of Microbiology, Monash University, Victoria, Australia; c School of Public Health, LKS Faculty of Medicine, The University of Hong Kong, Hong Kong, China; d HKU-Pasteur Research Pole, School of Public Health, LKS Faculty of Medicine, The University of Hong Kong, Hong Kong, China; e Department of Microbiology and Immunology, University of Melbourne at The Peter Doherty Institute for Infection and Immunity, Melbourne, Victoria, Australia; f Hoffmann-La Roche, Ltd., Basel, Switzerland; Icahn School of Medicine at Mount Sinai; University of Hong Kong

**Keywords:** antiviral resistance, ferret, virus, antiviral agents, influenza

## Abstract

Amino acid substitutions I38T and E23K in the influenza polymerase acidic (PA) protein lead to reduced susceptibility to the influenza antiviral drug baloxavir. The *in vivo* effectiveness of baloxavir and oseltamivir for treatment of these viruses is currently unknown. Using patient-derived influenza isolates, combination therapy was equally effective as monotherapy in reducing viral titers in the upper respiratory tract of ferrets infected with A(H1N1pdm09)-PA/E23K or A(H3N2)-PA/I38T. When treated with baloxavir plus oseltamivir, infection with a mixture of PA/I38T or PA/E23K and corresponding wild-type virus was characterized by a lower selection of viruses with reduced baloxavir susceptibility over the course of infection compared to baloxavir monotherapy. *De novo* emergence of the oseltamivir resistance mutation NA/H275Y occurred in ferrets treated with oseltamivir alone but not in ferrets treated with baloxavir plus oseltamivir. Our data suggest that combination therapy with influenza drugs with different mechanisms of action decreased the selection pressure for viruses with reduced drug susceptibility.

## INTRODUCTION

Antiviral drugs are important for the control of influenza, particularly for the treatment of patients who are hospitalized or for outpatients who are at a high risk for complications due to infection. Four neuraminidase inhibitors (NAIs) were licensed in the early 2000s; oseltamivir is the most commonly prescribed of these drugs (1). Baloxavir marboxil (referred to here as baloxavir) was licensed for the treatment of uncomplicated influenza in Japan and the United States in 2018. To date, baloxavir has been licensed in over 27 countries for the treatment of uncomplicated influenza and in some countries for those at high risk for complications or for postexposure prophylaxis. Baloxavir inhibits the polymerase acidic protein (PA) endonuclease function of the heterotrimeric influenza polymerase complex (2, 3).

The utility of an antiviral drug is lost or reduced if a virus acquires amino acid substitutions that decrease drug binding and result in reduced drug susceptibility; these have been identified for both the NAIs and baloxavir. For oseltamivir such substitutions are typically identified in 0.4 to 4% of posttreatment isolates from adults and in 3 to 37% of isolates from children (4); the NA/H275Y substitution is most common in A(H1N1pdm09) viruses and NA/E119V in A(H3N2) viruses (4). Amino acid changes at PA/I38 are associated with reduced susceptibility to baloxavir and have been identified following treatment in 2.3 to 9.7% of adults and 23% of children in separate phase III clinical trials (5, 6). PA/I38T is the most common substitution associated with reduced baloxavir susceptibility and is found at the highest frequency posttreatment in adolescents infected with A(H3N2) viruses. Other amino acid substitutions, including PA/E23K, PA/A37T, and PA/E119D, have been identified at lower frequencies posttreatment (<1% of patients) and are also associated with reduced susceptibility to baloxavir *in vitro*, but to a lower extent than PA/I38T (6–8). Due to viral fitness or drug selection pressure, the proportion of a variant in the total viral population can increase or decrease in frequency over the course of infection. Clinically, the emergence of viruses with reduced baloxavir susceptibility can also lead to a transient increase in viral titer and an increased duration of viral shedding (9). In two case studies, viruses with either PA/I38T and PA/E23K substitutions were detected in children who had not been treated with baloxavir but were household contacts of baloxavir-treated children; this suggests the potential for these viruses to transmit from person to person (10–12).

Combinations of antiviral drugs with different mechanisms of action have been used to reduce the emergence of drug-resistant viruses in patients with HIV or hepatitis C virus infection (13, 14). The availability of anti-influenza drugs with different mechanisms of action (such as oseltamivir and baloxavir) permits a consideration of combination therapy. The drugs are synergistic *in vitro*, and the combination of oseltamivir and baloxavir was more effective than oseltamivir monotherapy in reducing viral lung titers in a mouse model, even when treatment was delayed to 96 h postinfection (15, 16).

The effectiveness of baloxavir or oseltamivir monotherapy or a combination of both drugs against influenza viruses with amino acid substitutions associated with reduced susceptibility to baloxavir *in vitro* (e.g., PA/I38T and PA/E23K) is currently unknown. The primary aim of this study was to compare the effectiveness of oseltamivir and baloxavir combination therapy with either drug alone for the treatment of ferrets infected with wild-type (WT; drug-sensitive) influenza viruses or paired posttreatment isolates that contained either a PA/I38T or PA/E23K substitution that is known to reduce baloxavir susceptibility *in vitro*. We hypothesized that combination antiviral therapy would offer additional virological benefit over monotherapy for infection with influenza viruses with reduced susceptibility to baloxavir.

The secondary aim of the study was to use a mixed infection with baloxavir-sensitive variants with reduced susceptibility to model a scenario that could occur in patients, where a virus with reduced drug susceptibility may emerge and increase in relative proportion under drug treatment pressure. Ferrets were coinfected with the paired clinical isolates to determine the change in proportion of variant and WT viruses over the duration of viral shedding under selection pressure with drug monotherapy and combination therapy. We hypothesized that combination therapy would exert a lower selective pressure than baloxavir monotherapy on the relative proportion of viruses with reduced baloxavir susceptibility over time, since the viruses should remain sensitive to oseltamivir because it has a different mechanism of action.

To achieve these aims, antiviral treatment with baloxavir and oseltamivir was studied in ferrets infected with influenza viruses obtained from patients before and after treatment with baloxavir; one was an A(H3N2) PA/I38T variant, and the second was an A(H1N1pdm09) virus that contained a PA/E23K amino acid substitution.

## RESULTS

### *In vitro* drug susceptibility and drug interactions.

The two pairs of clinical isolates were tested for *in vitro* susceptibility to baloxavir and oseltamivir by determining the 50% effective concentration (EC_50_) and 50% inhibitory concentration (IC_50_), respectively. The FRA showed that A(H3N2)-PA/I38T and A(H1N1pdm09)-PA/E23K viruses had a 77- and 17-fold higher EC_50_ values for baloxavir compared to the corresponding pretreatment virus, respectively, and the yield reduction assay (YRA) showed 100- and 9-fold increases in EC_50_ compared to the corresponding WT. Confirming that the PA/E23K substitution results in a lower fold change reduction in baloxavir susceptibility than PA/I38T (Table 1). All four viruses were susceptible to the NAI drugs oseltamivir, zanamivir, peramivir, and laninamivir based on a neuraminidase enzyme inhibition assay (Table 1; see also Table S3 in the supplemental material).

**TABLE 1:** Influenza viruses utilized in study with *in vitro* susceptibility (EC_50_) and upper respiratory tract viral shedding characteristics in ferrets^*a*^

Parameter	A(H3N2)			A(H1N1pdm09)		
	100% WT	80% WT: 20% PA/I38T	100% PA/I38T	100% WT	80% WT: 20% PA/E23K	100% PA/E23K
Baloxavir EC_50_ (nM)						
FRA	0.6 ± 0.2	NA	46 ± 6.9	1.2 ± 0.2	NA	20.8 ± 10.2
YRA	0.4 ± 0.1	NA	40.1 ± 15.6	0.3 ± 0.02	NA	2.6 ± 0.07
Oseltamivir IC_50_ (nM)						
NIA	0.22 ± 0.2	NA	0.15 ± 0.11	0.3 ± 0.03	NA	0.33 ± 0.04
YRA	0.27 ± 0.2	NA	0.28 ± 0.19	0.5 ± 0.4	NA	0.61 ± 0.02
Duration of viral shedding (days)						
Placebo	6 ± 0	6.3 ± 0.5	6.3 ± 0.5	6.3 ± 0.5	6 ± 0	6 ± 0
Oseltamivir	5.3 ± 0.5	5.3 ± 0.5	3.7 ± 1.2	6 ± 0	6.7 ± 0.5	6.3 ± 0.5
Baloxavir	4.3 ± 0.9	4.7 ± 0.5	5.7 ± 0.5	4 ± 0.8	5.3 ± 0.5	6.3 ± 0.5
Combination	5 ± 0	3.7 ± 1.7	3.7 ± 0.9	4 ± 0.8	5.7 ± 1.2	5.7 ± 0.5
AUC						
Placebo	25.9 ± 1.2	25.1 ± 2.9	26 ± 0.7	30.8 ± 2.6	26.3 ± 1.2	27.9 ± 2.7
Oseltamivir	19.7 ± 2.7*	19.3 ± 1.8	16.5 ± 2.8*	29.8 ± 2.2	26.9 ± 1.7	26.2 ± 1.3
Baloxavir	18.7 ± 1.3**	18.6 ± 1.4	23.4 ± 1.4	21.8 ± 2.9*	24.7 ± 2.4	28.6 ± 0.8
Combination	20.4 ± 1.8**	16.8 ± 3.4	16.7 ± 3.4*	21.7 ± 2.2**	23.9 ± 3.3	25.7 ± 2.9

a Data are presented as means ± the standard deviations. *, *P* < 0.05; **, *P* < 0.01 (for the treatment group compared to placebo in area under the curve analysis [unpaired *t* test with Welch’s correction]). WT, wild type; NA, not applicable; FRA, focus reduction assay; NIA, neuraminidase inhibition assay; YRA, yield reduction assay; AUC, area under the curve.

10.1128/mbio.01056-22.6 TABLE S3 *In vitro* susceptibility of viruses to neuraminidase inhibitors. Download Table S3, PDF file, 0.6 MB. Copyright © 2022 Koszalka et al. 2022 Koszalka et al. https://creativecommons.org/licenses/by/4.0/ This content is distributed under the terms of the Creative Commons Attribution 4.0 International license.

**FIG 1:**
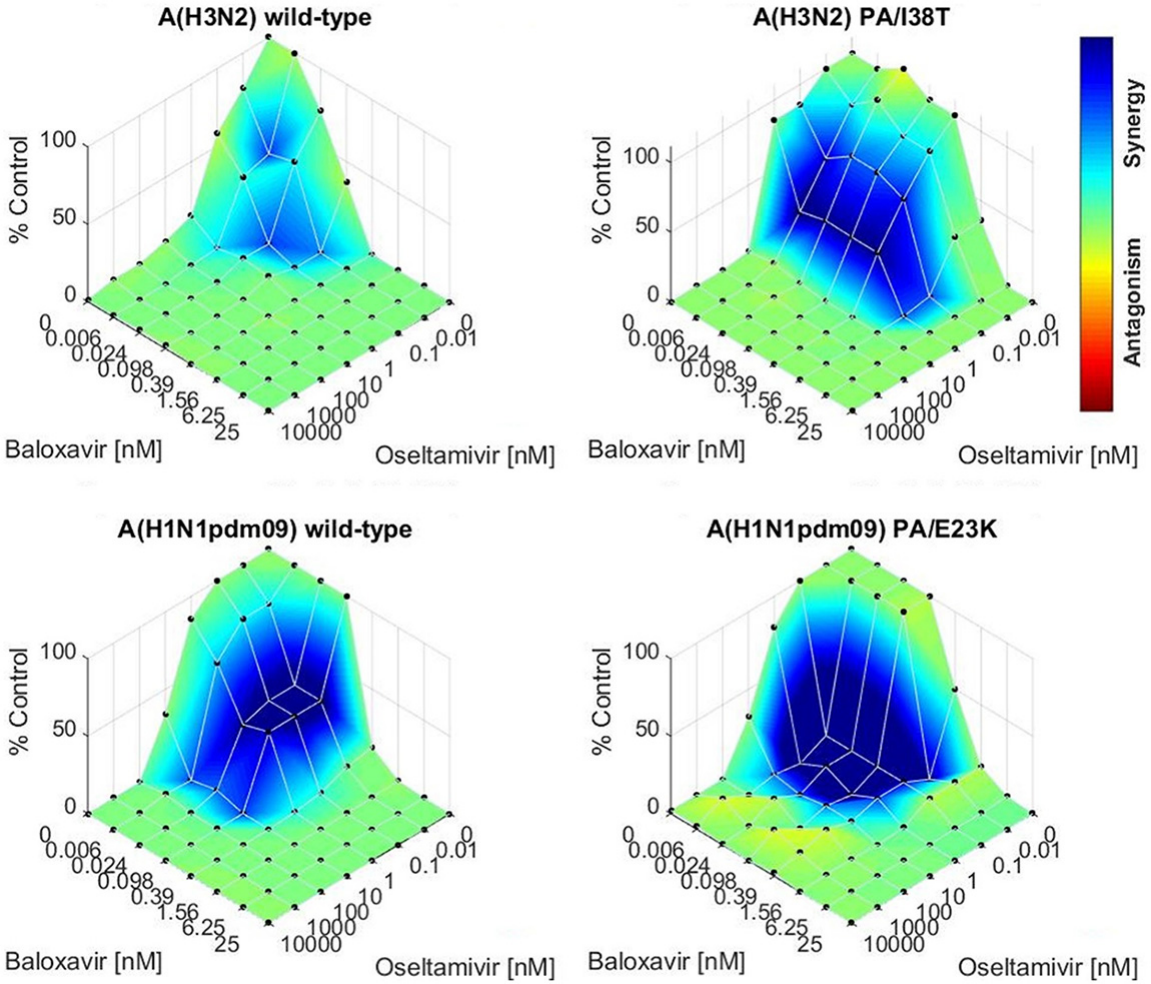
Surface plot to show interactions between baloxavir and oseltamivir against the growth of A(H3N2)-WT or PA/I38T and A(H1N1pdm09)-WT or PA/E23K viruses using the Bliss independence synergy model. The *z*-axis depicts the percent inhibition of viral growth relative to no drug (0 nM baloxavir plus 0 nM oseltamivir). The baloxavir and oseltamivir concentrations were tested in full factorial combination, and the colored shading on the plot depicts additive (green), synergistic (blue), and antagonistic (red) effects.

The viruses were tested *in vitro* against combinations of baloxavir and oseltamivir in a YRA to determine whether the drugs would be synergistic in their antiviral activity. Increasing concentrations of baloxavir and oseltamivir were analyzed alone and in combination; the synergistic potential was determined by the Bliss independence model. All four viruses were more effectively inhibited by a combination of baloxavir and oseltamivir compared to either drug alone (Fig. 1). Therefore, the drugs were synergistic even against viruses with reduced susceptibility to baloxavir. The greatest region of drug synergy occurred at low concentrations of either drug (0.006 to 1.56 nM baloxavir).

### Effectiveness of baloxavir and oseltamivir combination therapy in ferrets infected with WT viruses or viruses with reduced baloxavir susceptibility.

Ferrets infected with the pairs of clinical isolates were treated with either placebo, baloxavir or oseltamivir alone, or these agents in combination, and nasal washes were collected for 10 days to determine virus titers in the upper respiratory tract (see Fig. S1). An area-under-the-curve (AUC) analysis was performed to incorporate the viral titer and duration of viral shedding for ferrets in each antiviral treatment group.

10.1128/mbio.01056-22.1 FIG S1 Schematic of the experimental model used to assess baloxavir and oseltamivir combination therapy against influenza viruses with reduced antiviral susceptibility in ferrets. Ferrets were infected with 10^5^ TCID_50_/500 μL [A(H3N2) virus] or 10^4^ TCID_50_/500 μL [A(H1N1pdm09) virus] via the intranasal route, and antiviral treatment was commenced 24 h later. Antiviral treatment included 4 mL/kg placebo (subcutaneous single dose, methylcellulose vehicle), 10 mg/kg/day oseltamivir monotherapy (oral, BID), 4 mg/kg baloxavir monotherapy (subcutaneous, single dose), or a combination of baloxavir and oseltamivir (doses as described for each monotherapy). Nasal washes were collected daily for 10 days, and animal weights and temperatures were monitored for 14 days. On day 14, the ferrets were sacrificed, and a blood sample was obtained. Download FIG S1, PDF file, 0.6 MB. Copyright © 2022 Koszalka et al. 2022 Koszalka et al. https://creativecommons.org/licenses/by/4.0/ This content is distributed under the terms of the Creative Commons Attribution 4.0 International license.

**FIG 2:**
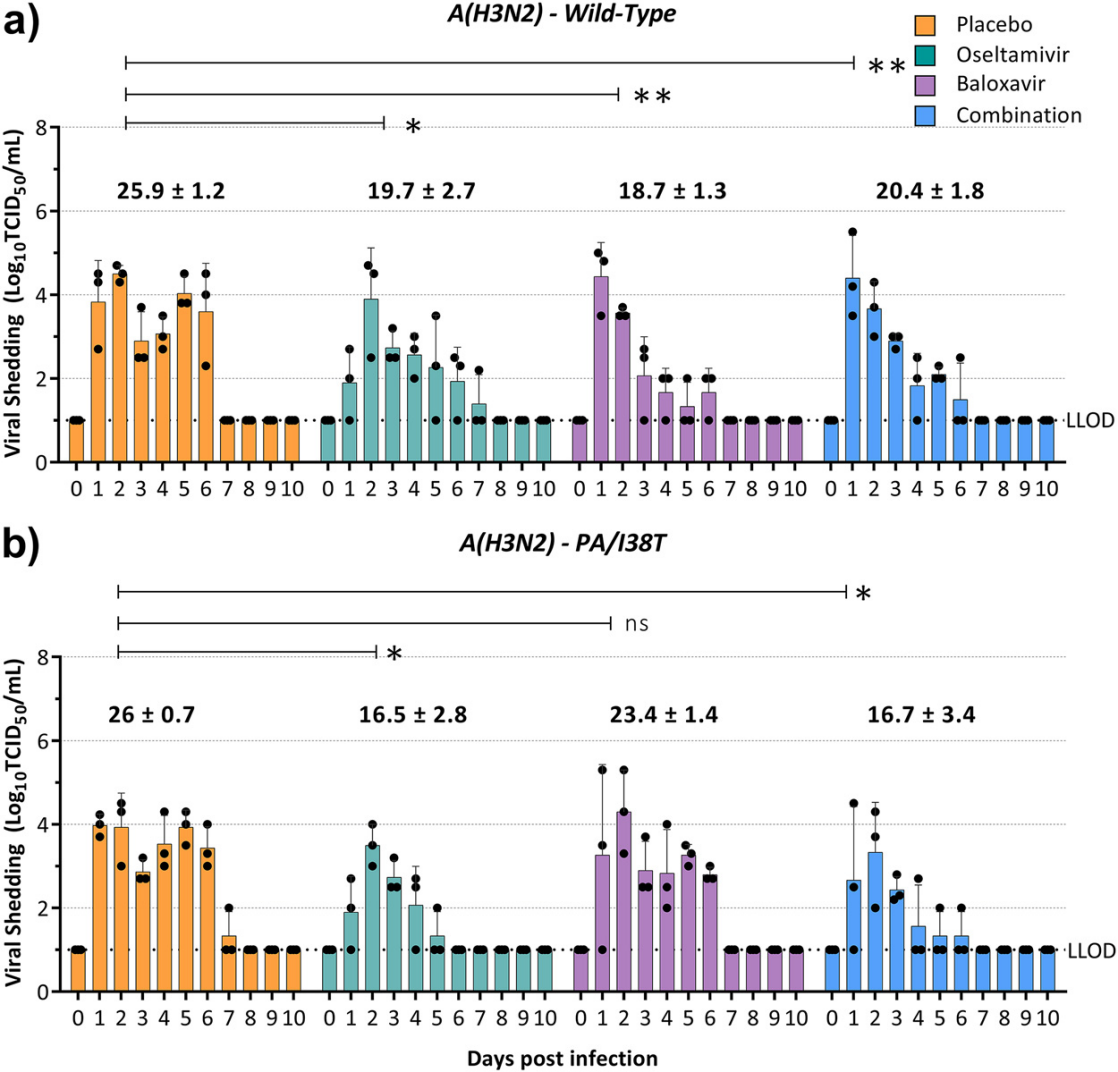
Effect of antiviral treatment on viral shedding in ferrets infected with A(H3N2)-WT or A(H3N2)-PA/I38T. Ferrets were inoculated intranasally with 10^5^ TCID_50_/500 μL with a pure population of A(H3N2)-WT (a) or a pure population of A(H3N2)-PA/I38T (b). Antiviral treatment was commenced 24 h postinfection with 4 mL/kg placebo (subcutaneous single dose, methylcellulose vehicle), 10 mg/kg/day oseltamivir monotherapy (oral, BID), 4 mg/kg baloxavir monotherapy (subcutaneous, single dose), or a combination of baloxavir and oseltamivir (doses as described for each monotherapy). Nasal washes were collected daily for 10 days, and the infectious virus titer was determined in MDCK-SIAT cells. The viral titers in the nasal washes of ferrets in each antiviral treatment group are represented as means ± the standard deviations, and the area under the curve for each group is shown above the bar graph. Statistical analysis was used to compare the area under the curve for each antiviral treatment to the corresponding placebo (unpaired *t* test with Welch’s correction; ns, nonsignificant; *, *P* < 0.05; **, *P* < 0.01). The LLOD for the assay is 10^1^ TCID_50_/mL.

**(i) A(H3N2)-wild-type and A(H3N2)-PA/I38T clinical isolate pair.** In ferrets infected with the A(H3N2)-WT virus, oseltamivir monotherapy (mean AUC ± the standard deviation [SD] = 19.7 ± 2.7, *P = *0.046) and combination treatment (AUC = 20.4 ± 1.8, *P < *0.01) reduced the AUC by 24 and 22% compared to the placebo (AUC = 25.9 ± 1.2), while baloxavir monotherapy (AUC = 18.7 ± 1.3, *P < *0.01) led to the greatest reduction in AUC relative to placebo (28%) (Fig. 2a). Combination treatment was not more effective at reducing viral shedding than either monotherapy. The duration of viral shedding was 1.7 days shorter in ferrets treated with baloxavir monotherapy (4.3 days) than in ferrets that received either oseltamivir monotherapy or combination treatment (5.3 and 5 days, respectively) or those that received placebo (6 days) (Table 1).

In ferrets infected with the A(H3N2)-PA/I38T virus, oseltamivir (AUC = 16.5 ± 2.8, *P = *0.03) and combination treatment (AUC = 16.7 ± 3.4, *P = *0.03) performed similarly and reduced the AUC by 37% and viral shedding by 2.7 days relative to the placebo (AUC = 26 ± 0.7) (Fig. 2b and Table 1), whereas baloxavir monotherapy (AUC = 16.5 ± 2.8, *P = *0.087) resulted in only a 10% reduction in AUC and a 0.6-day reduction in viral shedding, indicating that although some effect of baloxavir was retained, it was significantly reduced compared to that achieved against the WT virus (Fig. 2b).

**FIG 3:**
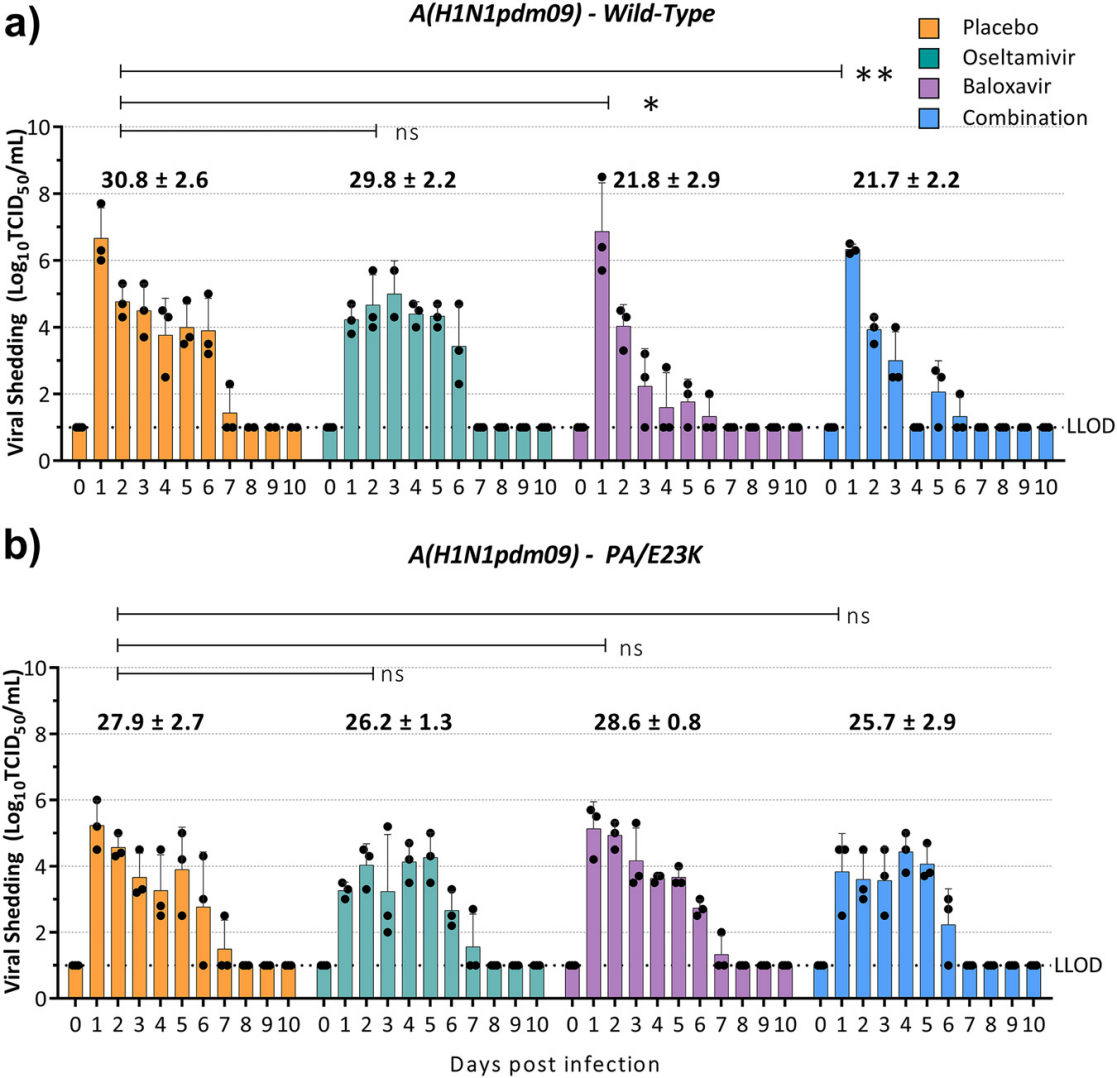
Effect of antiviral treatment on viral shedding in ferrets infected with A(H1N1pdm09)-WT or A(H1N1pdm09)-PA/E23K. Ferrets were inoculated intranasally with 10^4^ TCID_50_/500 μL with a pure population of A(H1N1pdm09)-WT (a) or a pure population of A(H1N1pdm09)-WT (b). Antiviral treatment was commenced 24 h postinfection with 4 mL/kg placebo (subcutaneous single dose, methylcellulose vehicle), 10 mg/kg/day oseltamivir monotherapy (oral, BID), 4 mg/kg baloxavir monotherapy (subcutaneous, single dose), or a combination of baloxavir and oseltamivir (doses as described for each monotherapy). Nasal washes were collected daily for 10 days, and infectious virus titers were determined in MDCK-SIAT cells. The viral titers in the nasal washes of ferrets in each antiviral treatment group are represented as means ± the standard deviations, and the area under the curve for each group is shown above the bar graph. Statistical analysis was used to compare the area under the curve for each antiviral treatment to the corresponding placebo (unpaired *t* test with Welch’s correction; ns, nonsignificant; *, *P* < 0.05; **, *P* < 0.01). The LLOD for the assay is 10^1^ TCID_50_/mL.

**(ii) A(H1N1pdm09)-WT and A(H1N1pdm09)-PA/E23K clinical isolate pair.** We sought to explore the *in vivo* effectiveness of baloxavir in ferrets infected with a clinical A(H1N1pdm09) isolate containing the PA/E23K substitution obtained after treatment, paired with a pretreatment isolate. In ferrets infected with the A(H1N1pdm09)-WT virus, baloxavir (AUC = 21.8 ± 2.9, *P = *0.01) and combination therapy (21.7 ± 2.2, *P < *0.01) reduced the average AUC by 29 and 30%, respectively, compared to the placebo (AUC = 30.8 ± 2.6) (Fig. 3a), and reduced the duration of viral shedding by an average of 2.3 days than placebo-treated animals (Table 1). Oseltamivir (AUC = 29.8 ± 2.2, *P = *0.21) treatment was less effective than baloxavir monotherapy or combination therapy in reducing viral shedding, with an AUC only 3% lower than in placebo-treated animals (Fig. 3a). This contrasts with the A(H3N2) virus, where oseltamivir monotherapy reduced the average AUC by 24% compared to the corresponding placebo group (Fig. 2a). A 1,000-fold reduction in mean viral titer was observed in baloxavir monotherapy-treated ferrets 1 day following antiviral treatment (Fig. 3a) compared to a 100-fold reduction in ferrets receiving placebo or combination treatment and no reduction in oseltamivir-treated ferrets. In addition, the NA/H275Y substitution was identified by pyrosequencing in all ferrets treated with oseltamivir monotherapy (3/3) but was not present in any other antiviral treatment group.

In ferrets infected with the A(H1N1pdm09)-E23K virus, a rapid reduction in viral load was not observed in the first 24 h following baloxavir treatment. In addition, the effectiveness of baloxavir monotherapy (AUC = 28.6 ± 0.8, *P = *0.59) was greatly reduced, with no significant difference in AUC compared to the placebo-treated group (AUC = 27.9 ± 2.7) (Fig. 3b), confirming that the A(H1N1pdm09)-PA/E23K amino acid substitution confers reduced susceptibility to baloxavir *in vivo*. Oseltamivir monotherapy (AUC = 26.2 ± 1.3, *P = *0.49) and combination therapy (AUC = 25.7 ± 2.9, *P = *0.39) resulted in average AUC reductions of only 6 and 7%, respectively, compared to the placebo (Fig. 3b). In these ferrets, the NA/H275Y amino acid substitution was present in all ferrets treated with oseltamivir monotherapy (3/3) 3 to 5 days after antiviral treatment was commenced and in one ferret treated with the oseltamivir-baloxavir combination (1/3) on the final day of infectious virus shedding.

### Effectiveness of baloxavir-oseltamivir combination therapy in ferrets infected with mixed populations of clinical isolates.

In patients, viruses with reduced susceptibility to baloxavir tend to emerge from a minor population that is selected for and increases in proportion over time during treatment. We modeled this situation by coinfecting ferrets with a 20:80 mixture of pre- and posttreatment isolates and studied the effectiveness of antiviral treatment on the competitive viral mixture estimated by pyrosequencing.

**FIG 4:**
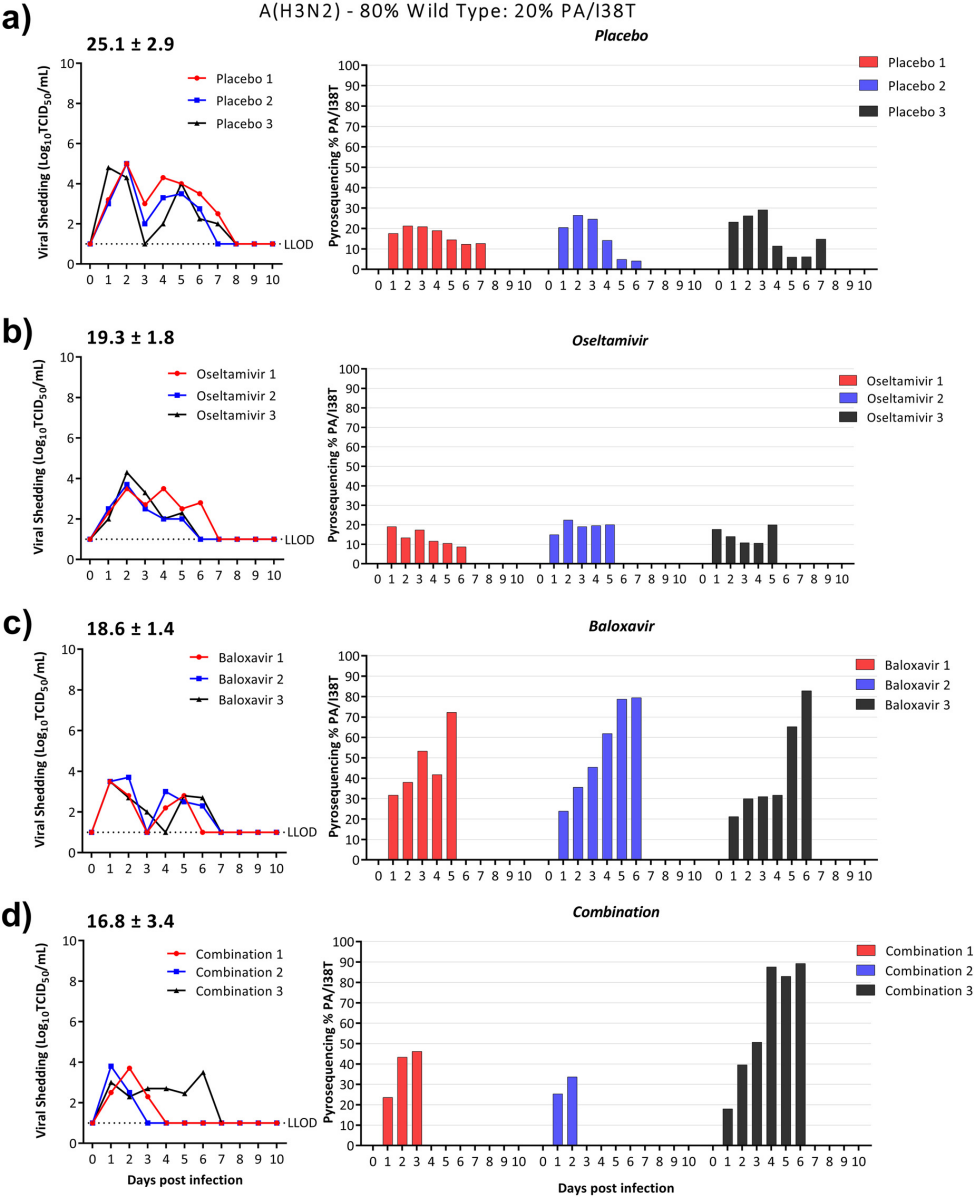
Effect of antiviral treatment on the relative proportion of A(H3N2)-PA/I38T in ferrets infected with a competitive mixture of WT:PA/I38T. Ferrets were intranasally inoculated with 10^5^ TCID_50_/500 μL of 20% A(H3N2)-PA/I38T:80% A(H3N2)-WT. Antiviral treatment was commenced 24 h postinfection with placebo (subcutaneous single dose, methylcellulose vehicle) (a), oseltamivir monotherapy (oral, BID) (b), baloxavir monotherapy (subcutaneous, single dose) (c), or combination therapy with oseltamivir and baloxavir (doses as described for each monotherapy) (d). Nasal washes were collected daily for 10 days. The infectious virus titers were determined by titration in MDCK cells (left panel), and the percentages of PA/I38T in the nasal wash were determined by pyrosequencing for the duration of viral shedding (right panel). The average area under the curve is represented above the viral shedding for each treatment group. The pyrosequencing plots are shown for each individual ferret, and the colors correspond to the virus titer in TCID_50_. Whole-genome sequencing was performed on samples obtained from day 5 or the final day of viral shedding. The LLOD for the TCID_50_ assay is 10^1^ TCID_50_/mL.

**(i) A(H3N2)-WT and A(H3N2)-PA/I38T clinical isolate pair.** In ferrets infected with a 20% A(H3N2)-PA/I38T:80% A(H3N2)-WT mixture, treatment with placebo or oseltamivir did not select for a significant increase (or decrease) in A(H3N2)-PA/I38T over the duration of viral shedding. However, the proportion of A(H3N2)-PA/I38T increased rapidly following baloxavir monotherapy to 72 to 83% on the final day of viral shedding (Fig. 4c). Even though baloxavir monotherapy increased the propensity of A(H3N2)-PA/I38T, both oseltamivir and baloxavir monotherapy reduced viral shedding to equivalent levels with 40% (AUC = 12.6 ± 1.4, *P = *0.065) and 39% (AUC = 13.3 ± 1.9, *P = *0.072) reductions in AUC, respectively, compared to placebo-treated ferrets (Fig. 4b and c).

In ferrets receiving combination treatment, two had a final proportion of 34 to 46%, A(H3N2)-PA/I38T, while the third ferret had a higher final proportion of 95%, which was comparable to the A(H3N2)-PA/I38T proportion in ferrets treated with baloxavir monotherapy (Fig. 4d). In these ferrets receiving combination treatment, the average duration of viral shedding was 2.7 days shorter than in placebo-treated ferrets (Table 1). The two ferrets with a low proportion of A(H3N2)-PA/I38T ceased shedding infectious virus on days 3 and 4 postinfection (days 2 and 3 after baloxavir treatment) (Fig. 4d), while the third ferret with a high proportion of A(H3N2)-PA/I38T had an extended duration of viral shedding that lasted 6 days postinfection (Fig. 4d). Viral shedding in ferrets treated with the combination of oseltamivir and baloxavir gave an average reduction of 34% (AUC = 16.8 ± 3.4, *P = *0.064) relative to placebo (AUC = 25.1 ± 2.9), but the difference did not reach statistical significance (Fig. 4a).

Whole-genome sequence (WGS) analysis was conducted on all ferrets to determine whether any additional amino acid substitutions were selected under antiviral pressure and to investigate whether the ferret receiving combination treatment with extended viral shedding had acquired any amino acid substitutions associated with reduced oseltamivir susceptibility. Several nonsynonymous amino acid changes were identified; of note, PA/E677K, NA/V240I, and NA/D251H were identified under combination treatment pressure (see Table S1). However, these substitutions did not lead to a phenotypic change in drug susceptibility (data not shown). We did not identify any amino acid changes to explain why the single ferret treated with the antiviral drug combination had extended viral shedding.

10.1128/mbio.01056-22.5 TABLE S2 Serum antibody hemagglutinin inhibition (HAI) responses 14 days postinfection. Download Table S2, PDF file, 0.6 MB. Copyright © 2022 Koszalka et al. 2022 Koszalka et al. https://creativecommons.org/licenses/by/4.0/ This content is distributed under the terms of the Creative Commons Attribution 4.0 International license.

**FIG 5:**
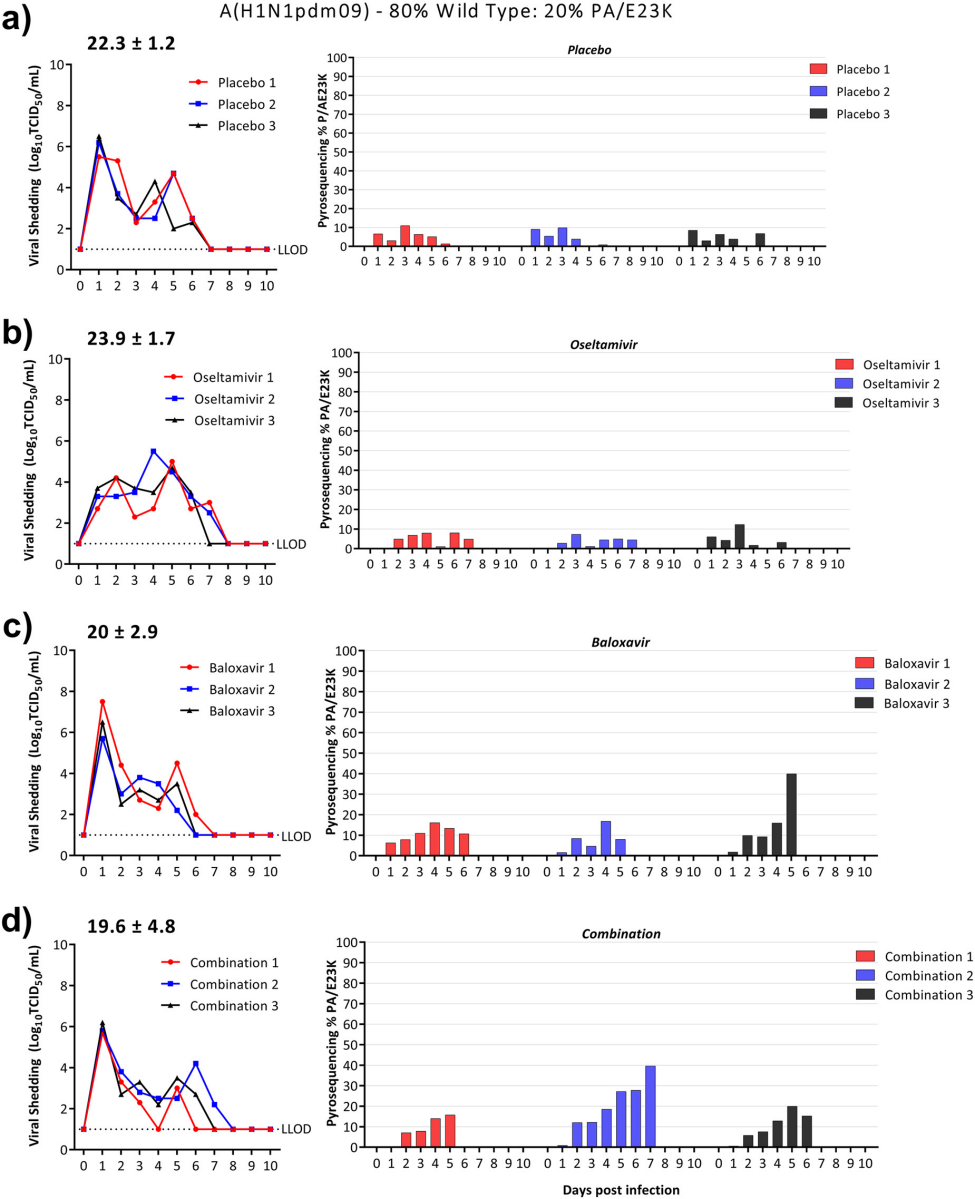
Effect of antiviral treatment on the relative proportion of A(H1N1pdm09)-PA/E23K in ferrets infected with a competitive mixture of WT and PA/E23K. Ferrets were intranasally inoculated with 10^4^ TCID_50_/500 μL of 20% A(H1N1pdm09)-PA/E23K:80% A(H1N1pdm09)-WT. Antiviral treatment was commenced 24 h postinfection with placebo (subcutaneous single dose, methylcellulose vehicle) (a), oseltamivir monotherapy (oral, BID) (b), baloxavir monotherapy (subcutaneous, single dose) (c), or combination therapy with oseltamivir and baloxavir (doses as described for each monotherapy) (d). Nasal washes were collected daily for 10 days. The infectious virus titers were determined by titration in MDCK cells (left panel), and the percentages of PA/I38T in the nasal wash were determined by pyrosequencing for the duration of viral shedding (right panel). The average area under the curve is represented above the viral shedding for each treatment group. The pyrosequencing plots are shown for each individual ferret, and the colors correspond to the TCID_50_ shedding. Whole-genome sequencing was performed on samples obtained from day 5. The LLOD for the TCID_50_ assay is 10^1^ TCID_50_/mL.

**(ii) A(H1N1pdm09)-WT and A(H1N1pdm09)-PA/E23K clinical isolate pair.** Treatment with placebo or oseltamivir did not select for a significant increase (or decrease) in viruses that contained PA/E23K over the duration of viral shedding. For ferrets treated with baloxavir monotherapy, the proportion of A(H1N1pdm09)-PA/E23K ranged from 8 to 40% on the final day of virus shedding (Fig. 5c). In ferrets treated with the combination, the proportion of A(H1N1pdm09)-PA/E23K ranged from 15 to 40%, values that were similar to that seen for baloxavir monotherapy (Fig. 5d). Even though the overall duration of virus shedding for the A(H1N1pdm09)-PA/E23K mixture was longer than for the A(H3N2)-PA/I38T mixture, thereby allowing a longer duration for selection of viruses with reduced antiviral susceptibility, the proportion of the A(H1N1pdm09)-PA/E23K was not as strongly selected as A(H3N2)-PA/I38T was in ferrets in the competitive mixture experiments. Baloxavir monotherapy and combination therapy resulted in similarly minor reductions in AUC of 6% (AUC = 24.7 ± 2.4, *P = *0.31) and 9% (AUC = 23.9 ± 3.3, *P = *0.43), respectively, compared to the placebo treatment, even in the presence of A(H1N1pdm09)-PA/E23K (AUC = 26.3 ± 1.2) (Table 1).

WGS was performed on samples obtained from ferrets with a mixed infection on day 5 for genetic analysis. Three PA substitutions—S272R, P325S, and R496Q—were identified in the combination-treated ferrets. All three ferrets treated with oseltamivir monotherapy had viruses with an NA/H275Y substitution which is known to reduce susceptibility to oseltamivir (see Table S1). This was associated with prolonged viral shedding in ferrets in the oseltamivir treatment group, with an AUC that was 7% greater than placebo (AUC = 23.9 ± 1.7, *P = *0.27) (Fig. 5b). In the combination-treated ferrets, the NA/H275Y substitution was only identified in one ferret on the final day of infectious viral shedding (1/3). There were no NA/H275Y substitutions identified in placebo-treated ferrets or ferrets treated with baloxavir alone.

10.1128/mbio.01056-22.4 TABLE S1 Summary of amino acid substitutions identified from whole-genome sequencing in mixed-infection ferrets treated with placebo, oseltamivir, baloxavir, or a combination of oseltamivir and baloxavir. Download Table S1, PDF file, 0.4 MB. Copyright © 2022 Koszalka et al. 2022 Koszalka et al. https://creativecommons.org/licenses/by/4.0/ This content is distributed under the terms of the Creative Commons Attribution 4.0 International license.

## DISCUSSION

The effectiveness of baloxavir and oseltamivir combination therapy was evaluated on pairs of influenza clinical isolates that have reduced *in vitro* susceptibility to baloxavir due to two different amino acid substitutions, PA/I38T and PA/E23K; we also tested whether combination drug treatment could reduce the selection of these viruses. To our knowledge, this study is the first to assess the PA/E23K substitution *in vivo* and the first to study the effectiveness of baloxavir against viruses with either substitution (A(H3N2)-PA/I38T and A(H1N1pdm09)-PA/E23K) in the ferret model.

Our results demonstrate *in vitro* drug synergy for the combination of oseltamivir and baloxavir, even against viruses with reduced baloxavir susceptibility. However, the *in vivo* findings were discordant from the *in vitro* findings; treatment of baloxavir-sensitive viruses with the oseltamivir-baloxavir combination *in vivo* did not offer significant benefits over monotherapy. We confirmed that baloxavir monotherapy provides only a minor benefit in the reduction of viral shedding for ferrets infected with PA/I38T *in vivo* and that baloxavir-oseltamivir provided no added benefit over monotherapy. Baloxavir was also not effective in reducing viral shedding for the PA/E23K virus relative to the placebo treatment. As in our study, the PA/I38T substitution leads to a significant reduction of baloxavir effectiveness. Furthermore, baloxavir monotherapy provided only a small reduction in AUC for the A(H3N2)-PA/I38T virus and no effect on the A(H1N1pdm09)-PA/E23K virus, although the *in vitro* baloxavir EC_50_ is 4.5-fold lower for PA/E23K than for PA/I38T. It is known that A(H1N1pdm09) viruses tend to replicate more efficiently than A(H3N2) viruses in ferrets; this may result in a greater “barrier” for antiviral activity against the A(H1N1pdm09) virus (17).

For oseltamivir monotherapy, previous studies have not shown an antiviral effect in a treatment model (i.e., when treatment is commenced after viral infection); alternative study designs such as prophylactic oseltamivir treatment or donor/recipient viral transmission models have been used (18–21). Our study design recapitulates a more realistic clinical scenario than providing drug at the time of (or prior to) infection but resulted in low oseltamivir effectiveness against A(H1N1pdm09) viruses.

*In vitro* studies, including ours, have shown that the combination of baloxavir and oseltamivir is synergistic (22). However, synergy has not been demonstrated in mice or ferrets. Mice infected with influenza A/PR/8/34 showed equal reductions in viral lung titer after combination treatment with baloxavir (0.5 mg/kg, twice daily [BID]) and oseltamivir (10 mg/kg, BID) compared to baloxavir monotherapy alone (16). In an A(H3N2) immunodeficient mouse model, the reduction in lung viral titer was the same in mice treated with baloxavir monotherapy (40 mg/kg, single dose) as for mice treated with baloxavir-oseltamivir (40 mg/kg [single dose] and 20 mg/kg [BID], respectively) (23). For a virus that harbors a PA/I38T or PA/E23K substitution, treatment is unlikely to provide a significant virological benefit to patients; oseltamivir monotherapy or oseltamivir-baloxavir combination therapy may be useful, but only if oseltamivir is effective (5, 24). This suggests that alternative treatment options maybe be required to treat a virus that contains a PA/E23K substitution.

Combination treatment reduced the rapid selection of A(H3N2)-PA/I38T and was virologically more effective than baloxavir monotherapy in two of the three ferrets in the mixed viral infections. Notably, A(H3N2)-PA/I38T increased to a high proportion following baloxavir monotherapy, but the reduction in viral shedding was similar to oseltamivir monotherapy and lower than for the placebo. There was a lower propensity to select for A(H1N1pdm09)-PA/E23K compared to A(H3N2)-PA/I38T under baloxavir selection pressure (either monotherapy or combination therapy) and, interestingly, the *de novo* selection of NA/H275Y was greatly reduced with combination therapy compared to oseltamivir monotherapy. A small increase in the proportion of A(H1N1pdm09)-PA/E23K still led to reduced viral shedding with baloxavir monotherapy and combination therapy relative to the placebo.

Baloxavir is administered as a single dose, and further adjustments to the antiviral treatment course may not be feasible if a virus with reduced susceptibility to baloxavir emerges in a patient. Our study suggests that although the proportion of resistant virus may increase over time in an otherwise-healthy host, it may not lead to a significant change in the duration and amplitude of viral shedding. On the other hand, prolonged viral shedding in immunocompromised individuals increases the risk of selecting resistant viruses during antiviral treatment; several case reports have described this phenomenon for the NAIs (25–27). Investigators may alter antiviral drug treatment regimens if variants emerge in immunocompromised patients, but in otherwise-healthy hosts a change in antiviral treatment may have negligible benefit, particularly if the duration of viral shedding is short (28, 29). Combination therapy may be useful from the outset of treatment since our results suggest that baloxavir-oseltamivir may be effective in decreasing the selection of viruses with reduced susceptibility. However, if a substitution such as PA/E23K is present in the initial infection, no antiviral treatment is likely to provide a virological benefit. A serial passaging study in mice has also shown that baloxavir-oseltamivir can reduce the *de novo* emergence of variants with reduced susceptibility to either drug (30).

A phase III clinical trial in hospitalized patients with influenza (Flagstone, NCT03684044) showed that the addition of baloxavir to the “standard of care” oseltamivir compared to the placebo did not affect the time to clinical improvement: 97.5 and 100.2 h, respectively. The time to cessation of viral shedding was reduced by 39.8 h in the NAI-baloxavir-treated group (23.9 h) compared to the NAI-placebo-treated group (63.7 h) (31). In patients infected with an A(H3N2) virus, no PA/I38X or NA/H275Y substitutions were identified following NAI-baloxavir treatment. For A(H1N1pdm09), a PA/I38X variant was selected in 2% of patients (3/134) treated with NAI-baloxavir and NA/H275Y occurred in 2.5% of patients (5/199) treated with an NAI (combined with either placebo or baloxavir). Notably, combination treatment led to a dual amino acid change (NA/H275Y and PA/I38T) in two immunocompromised patients. In previous trials, monotherapy with baloxavir or oseltamivir for A(H1N1pdm09) viruses, resulted in 9.7 and 5% of posttreatment isolates, respectively, with PA/I38X or NA/H275Y. Therefore, in the Flagstone trial, combination treatment in a hospitalized treatment group had an overall lower selection of viruses with reduced susceptibility and a more rapid reduction of viral shedding, but the emergence of variants with reduced susceptibility to both drugs in two patients is of concern (31).

Viral fitness is a consideration since it influences the likelihood that a virus will transmit efficiently from person to person. Studies on the fitness of viruses with PA/I38X vary, but the fitness tends to be similar to matched baloxavir-sensitive viruses in animal models (32–35). We continued the analysis of a previously studied clinical isolate pair, where it was determined that the amino acid substitution A(H3N2)-PA/I38T results in some within-host attenuation of viral fitness in ferrets (36). Placebo-treated ferrets A(H1N1pdm09)-PA/E23K virus undergo a small reduction in AUC relative to the WT, suggesting that this substitution may reduce viral fitness. Additional *in vivo* studies are needed to determine the fitness of viruses with substitutions in the PA gene other than PA/I38X.

In conclusion, we have shown here that amino acid substitutions PA/I38T and PA/E23K lead to a reduction on baloxavir effectiveness *in vivo* and that baloxavir and oseltamivir, used in combination, do not result in a synergistic benefit. We did, however, show that combination treatment reduced the selection of A(H3N2)-PA/I38T viruses relative to baloxavir monotherapy. Given that such variants are of significant concern, further investigations of combination treatment with baloxavir and oseltamivir as a method to reduce the selection of viruses are warranted.

## MATERIALS AND METHODS

### Cells.

Madin-Darby canine kidney (MDCK)-SIAT1 cells were cultured at 37°C and 5% CO_2_ in Dulbecco modified Eagle medium (DMEM; Gibco, USA). The DMEM was supplemented with 10% fetal bovine serum (Bovogen Biologicals, Australia), 1× GlutaMAX (Gibco), 1× minimal essential medium (MEM) nonessential amino acid solution (Gibco), 0.06% sodium bicarbonate (Gibco), 20 μM HEPES (Gibco), 100 U/mL penicillin-streptomycin solution (Gibco), and 1 mg/mL Geneticin (Gibco).

### Antiviral compounds.

Shionogi & Co., Ltd., synthesized and kindly provided baloxavir acid, the active form of baloxavir marboxil, for these studies. For the *in vitro* experiments, baloxavir acid was prepared in dimethyl sulfoxide (Sigma-Aldrich, USA), filtered with a 0.2-μm surfactant-free cellulose acetate filter (Thermo Fisher, USA), and stored in aliquots at −80°C at a concentration of 20 mM. For the *in vivo* studies, a 1-mg/mL suspension of baloxavir acid was prepared in 0.5% (wt/vol) methylcellulose (Sigma-Aldrich, Australia), immediately prior to administration in ferrets (37). For the *in vivo* studies, oseltamivir phosphate (Thermo Fisher, USA) was prepared at 10 mg/mL by dilution in sterile 0.5% (vol/vol) sugar/phosphate-buffered solution (PBS) solution.

### Viruses.

The clinical isolate pairs were kindly provided by Shionogi & Co. and obtained from nasopharyngeal swabs in patients enrolled in the phase III clinical trial (CAPSTONE-1, NCT02954354; BLOCKSTONE, JapicCTI-184180). The first clinical isolate pair is termed “344103” and was previously described (9, 36). The pretreatment isolate is the “wild type” (WT; i.e., lacking a PA/I38T substitution), and the posttreatment isolate contains a PA/I38T substitution. The second clinical isolate pair, termed PNA508 and PNA012, consists of a pre- and posttreatment isolate that contained a PA/E23K substitution (7). PA, NA, and HA genes in the clinical isolate pairs were Sanger sequenced and contained no other amino acid substitutions. The viruses were propagated from nasopharyngeal swabs in MDCK-SIAT1 cells.

### *In vitro* characterization of viruses.

The sensitivity of pre- and posttreatment isolates to baloxavir was determined by a focus reduction assay, as previously described (38). The susceptibility of viruses was determined by measuring the concentration of baloxavir acid required to reduce viral focus-forming units by 50% (EC_50_). The percentage of inhibition at each concentration of baloxavir acid was determined and analyzed by nonlinear regression analysis (GraphPad Prism, USA). The sensitivity of the isolates to neuraminidase inhibitor drugs was determined by a fluorometric NA inhibition assay with the substrate 2′-(4-methylumbelliferyl)-a-d-*N*-acetylneuraminic acid (MUNANA; Sigma-Aldrich, Castle Hill, NSW, Australia). The enzymatic activity of oseltamivir carboxylate was determined as previously described (39). The NAI concentration that inhibited 50% of NA activity (IC_50_) was determined using the JASPR (version 1.2; CDC, USA) software program.

### *In vitro* synergy experiments.

Synergy was determined with a yield reduction assay, since the effect of oseltamivir is not reliably measured by focus reduction assay. To perform the yield reduction assay, MDCK-SIAT cells were seeded into a 96-well plate as described above. Cell monolayers were washed with PBS and infected with 100 virus particles (calculated based on virus titer determined by TCID_50_) for 90 min. The viral inoculum was then removed, and the cells were overlaid with baloxavir (0 to 25 nM, 4-fold serial dilution) or oseltamivir (0 to 10,000 nM, 10-fold serial dilution) alone or in full factorial combination. The plates were incubated for 24 h at 35°C, and the supernatant was collected and quantified to determine the infectious viral titer by using a TCID_50_ assay.

Synergy was determined using Combenefit software, the viral titer was expressed as the percent inhibition compared to a no-drug control (0 nM baloxavir, 0 nM oseltamivir). The software scores the percent inhibition difference between the single-drug titer and inhibition by the combined drug. If the reduction in titer is greater than that of either drug alone, the combination is synergistic.

### Ethics statement.

Ferret experiments were conducted under the guidelines of the University of Melbourne Biochemistry & Molecular Biology, Dental Science, Medicine, Microbiology & Immunology, and Surgery Animal Ethics Committee, in accordance with the NHMRC Australian code of practice for the care and use of animals for scientific purposes (8th edition). These experiments are listed under AEC 20033.

### Ferrets.

Outbred male and female ferrets (Mustela putorius furo) more than 6 months old were obtained from independent vendors. Ferrets weighed a minimum of 600 g, seronegativity to recently circulating influenza viruses was tested by a hemagglutination inhibition assay prior to study commencement. Ferrets were provided with *ad libitum* food and water for the duration of the experimental period.

### Infection of ferrets.

Experiments were organized into two series. The first series utilized the A(H3N2) clinical isolate pair, and the second series utilized the A(H1N1pdm09) clinical isolate pair. Virus stocks for ferret inoculation were diluted to 10^4^ and 10^5^ TCID_50_/500 μL in PBS for the A(H1N1pdm09) and A(H3N2) clinical isolate pairs, respectively. For the 100% WT pretreatment isolate and 100% posttreatment isolate, pure populations of virus were used for infection. In coinfected ferrets (20% variant:80% WT), the mixtures were prepared based on the TCID_50_ titer. A total of 36 ferrets were used in each experiment (72 ferrets for the complete study) with 12 ferrets in each infection group: 100% WT, 100% variant, or 20% variant:80% WT. For viral inoculation, the ferrets were given a reversible anesthesia via an intramuscular injection using a mixture of ketamine (10 mg/kg; Troy Laboratories), midazolam (0.5 mg/kg; Troy Laboratories), and medetomidine (0.02 mg/kg; Troy Laboratories) that was antagonized following the procedure by atipamezole (0.01 mg/kg; Troy Laboratories). During anesthesia, the ferrets were inoculated via the intranasal route with 250 μL of virus suspension in each nostril.

### Antiviral treatment of ferrets.

Three of the twelve ferrets were each assigned to placebo treatment, baloxavir monotherapy, oseltamivir monotherapy, or baloxavir-oseltamivir combination therapy group. All antiviral treatment was commenced at 24 h postinfection. Baloxavir treatment was administered as previously described (37). Briefly, a single dose was delivered under reversible anesthesia. The baloxavir acid suspension was administered by four subcutaneous injections on the dorsal region (1 mg/kg per site; 4 mg/kg baloxavir acid per ferret). Placebo-treated ferrets received a single dose of methylcellulose as a subcutaneous injection on the dorsal region (1 mL/kg per site; 4 mL/kg methyl cellulose per ferret). Oseltamivir treatment was administered orally for 5 days at 5 mg/kg BID in nonsedated ferrets (10 mg/kg/day). Combination-treated ferrets were administered a single dose of baloxavir acid and oseltamivir phosphate for 5 days BID, as described above. Treatment doses are based on prior pharmacokinetic analyses that achieved plasma concentration-time curves similar to those that occur in humans (37, 40).

### Ferret monitoring and sample collection.

The body temperatures and weights of the ferrets were measured daily (Fig. S2 and S3). Nasal washes were collected daily for 10 consecutive days from sedated ferrets (intramuscular injection of xylazine at 5 mg/kg) by instilling 1 mL of sterile PBS through the nostril. Ferrets were sacrificed at 14 days postinfection; anesthesia was administered by intramuscular injection (ketamine [≥25 mg/kg] and xylazine [≥5 mg/kg]), followed by an overdose of pentobarbitone sodium (Lethabarb; 0.5 mL/kg). Blood was collected by cardiac puncture 14 days postinfection, and the antibody response to a recently circulating virus that matched the viral subtype was measured by a hemagglutination inhibition assay (Table S2). Two ferrets were sacrificed on days 7 and 8 postinfection under the guidance of the animal ethics committee [ferret 2, baloxavir treated, A(H3N2)-PA/I38T; ferret 3, placebo treated, A(H1N1pdm09)-WT].

10.1128/mbio.01056-22.2 FIG S2 Summary of percent change in weight and temperature of ferrets infected with the A(H3N2) clinical isolate pair compared to the starting baseline. The weights and temperatures of ferrets were measured daily, and the line plot depicts the mean and standard deviation in each ferret treatment group. Download FIG S2, PDF file, 0.7 MB. Copyright © 2022 Koszalka et al. 2022 Koszalka et al. https://creativecommons.org/licenses/by/4.0/ This content is distributed under the terms of the Creative Commons Attribution 4.0 International license.

10.1128/mbio.01056-22.3 FIG S3 Summary of changes in percent weights and percent temperatures of ferrets infected with the A(H1N1pdm09) clinical isolate pair compared to the starting baseline. The weights and temperatures of ferrets were measured daily, and the line plot depicts the means and standard deviations for each ferret treatment group. Download FIG S3, PDF file, 0.7 MB. Copyright © 2022 Koszalka et al. 2022 Koszalka et al. https://creativecommons.org/licenses/by/4.0/ This content is distributed under the terms of the Creative Commons Attribution 4.0 International license.

### Virological analysis.

The infectious viral titer was determined by titration in MDCK-SIAT cells (lower limit of detection [LLOD] at 2 log_10_ TCID_50_/mL), as previously described (41). Viral RNA was extracted from nasal wash samples with a NucleoMag VET isolation kit (Macherey-Nagel) on a KingFisher Flex (Thermo Fisher Scientific) platform according to the manufacturer’s instructions. For pyrosequencing analysis, primers and reaction conditions described earlier (42) were utilized for the PA/I38T substitution. For the PA/E23K substitution, PCR amplification was performed with specific primers (forward, GCTTCAATCCAATGATCGTC; reverse, 5′-biotin-CATGAAACAAACTTCCAAATGTG). Pyrosequencing was performed with a TGCGGAAAAGGCAATGAA primer. For the NA/H275Y substitution in influenza A(H1N1pdm09) virus, the primers and reaction conditions were as previously described (43). The lower and upper limits of detection for pyrosequencing analysis were 5 and 95%, respectively, of the variant.

### Whole-genome sequencing.

Nasal wash samples from day 5 postinfection (or the final day of viral shedding) in coinfected ferrets (20% variant:80% WT) were further analyzed by whole-genome sequencing. The samples were passaged once in cell culture prior to sequencing to ensure the viral load was sufficient for WGS. Viral RNA was extracted with a QIAamp viral RNA minikit (Qiagen), and one-step RT-PCR was performed with universal influenza A primers using the qSCRIPT XLT one-step kit (Quanta). The polymerase genes PB2, PB1, and PA were amplified in separate reactions to ensure gene coverage. Sequencing libraries were generated using a Nextra library preparation kit, and sequencing was performed using Illumina iSeq.

### Statistical analysis.

Data analysis was performed using Prism (v6; GraphPad Software). The AUC for TCID_50_ was compared by using an unpaired Student *t* test with Welch’s correction. Samples below the LLOD were assigned zero values for graphing and statistical analyses. A *P* value of <0.05 was considered statistically significant.
