# Supplementary material for: Effect of Baloxavir and Oseltamivir in Combination on Infection with Influenza Viruses with PA/I38T or PA/E23K Substitutions in the Ferret Model
Source: mBio. 2022 Aug 8;13(4):e01056-22. doi: 10.1128/mbio.01056-22 (PMC9426601; doi:10.1128/mbio.01056-22)
Supplement: TABLE S3 [file mbio.01056-22-s0006.pdf]

Supplementary Table S3. In vitro susceptibility of viruses to neuraminidase inhibitors

Virus	Neuraminidase Inhibitor* Zanamivir IC_{50} (nM)	Oseltamivir IC_{50} (nM)	Peramivir IC_{50} (nM)	Laninamivir IC_{50} (nM)
**A(H3N2)-WT**	0.57 ± 0.01	0.22 ± 0.01	0.19 ± 0.01	0.96 ± 0.02
**A(H3N2)-PA/I38T**	0.36 ± 0.3	0.15 ± 0.11	0.12 ± 0.1	0.62 ± 0.5
**A(H1N1pdm09)-WT**	0.33 ± 0.04	0.3 ± 0.03	0.15 ± 0.03	0.41 ± 0.015
**A(H1N1pdm09)-PA/E23K**	0.39 ± 0.03	0.33 ± 0.04	0.17 ± 0.01	0.46 ± 0.02

***** IC_{50} values represented as Mean ± Standard Deviation, results from three independent experiments
