# Supplementary material for: Effect of Baloxavir and Oseltamivir in Combination on Infection with Influenza Viruses with PA/I38T or PA/E23K Substitutions in the Ferret Model
Source: mBio. 2022 Aug 8;13(4):e01056-22. doi: 10.1128/mbio.01056-22 (PMC9426601; doi:10.1128/mbio.01056-22)
Supplement: FIG S1 [file mbio.01056-22-s0001.pdf]

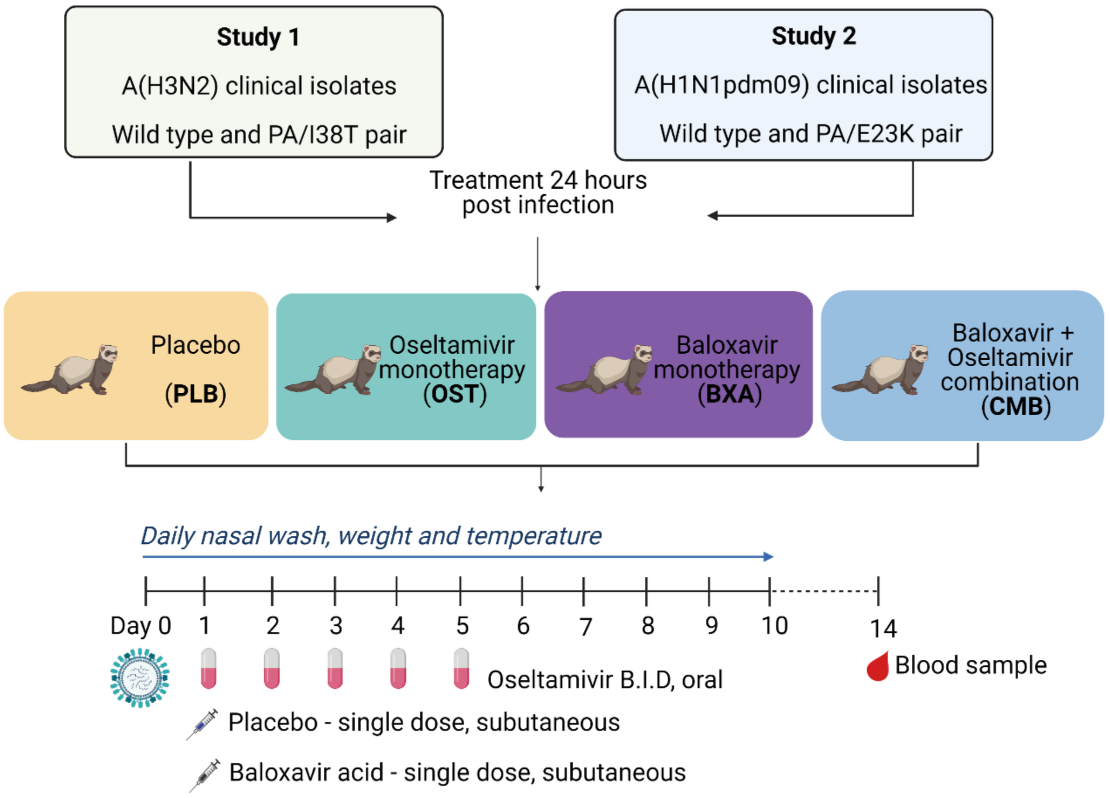

Supplementary Figure S1. Schematic of the experimental model used to assess baloxavir and oseltamivir combination therapy against influenza viruses with reduced antiviral susceptibility in ferrets.

Ferrets were infected with 10^5 $TCID_{50}$/500 µL (A(H3N2) virus) or 10^4 $TCID_{50}$/500 µL (A(H1N1pdm09) virus) via the intranasal route and antiviral treatment was commenced 24 hours later. Antiviral treatment included 4 mL/kg placebo (subcutaneous single dose, methylcellulose vehicle), 10 mg/kg/day oseltamivir monotherapy (oral, twice a day BID), 4 mg/kg baloxavir monotherapy (subcutaneous, single dose) or a combination of baloxavir and oseltamivir (doses as described for each monotherapy). Nasal washes were collected daily for 10 days and animal's weight and temperature were monitored for 14 days. On day 14 ferrets were sacrificed and a blood sample was obtained.
