# Supplementary material for: Effect of Baloxavir and Oseltamivir in Combination on Infection with Influenza Viruses with PA/I38T or PA/E23K Substitutions in the Ferret Model
Source: mBio. 2022 Aug 8;13(4):e01056-22. doi: 10.1128/mbio.01056-22 (PMC9426601; doi:10.1128/mbio.01056-22)
Supplement: TABLE S2 [file mbio.01056-22-s0005.pdf]

Supplementary Table S2. Serum antibody hemagglutinin inhibition (HAI) responses 14 days post-infection

	**Treatment group**			
Virus/Ferret number	**Placebo**	**Oseltamivir**	**Baloxavir**	**Combination**
**A(H3N2)-WT**				
Ferret 1	160	320	160	160
Ferret 2	160	160	80	80
Ferret 3	80	320	160	160
**80% A(H3N2)-WT : 20% A(H3N2)-PA/I38T**				
Ferret 1	160	80	640	80
Ferret 2	160	80	160	160
Ferret 3	160	320	160	160
**A(H3N2)-PA/I38T**				
Ferret 1	320	160	320	320
Ferret 2	160	320	80*	320
Ferret 3	160	160	160	320
**A(H1N1pdm09)-WT**				
Ferret 1	1280	2560	1280	1280
Ferret 2	2560	2560	1280	2560
Ferret 3	320**	2560	2560	2560
**80% A(H1N1pdm09)-WT : 20% A(H1N1pdm09)-PA/E23K**				
Ferret 1	2560	2560	2560	640
Ferret 2	1280	2560	2560	2560
Ferret 3	1280	2560	1280	1280
**A(H1N1pdm09)-PA/E23K**				
Ferret 1	2560	1280	2560	2560
Ferret 2	1280	640	1280	2560
Ferret 3	1280	1280	1280	2560

*Ferret culled and cardiac bleed obtained on day 8 post-infection

** Ferret culled and cardiac bleed obtained on day 7 post-infection
