# Supplementary material for: Effect of Baloxavir and Oseltamivir in Combination on Infection with Influenza Viruses with PA/I38T or PA/E23K Substitutions in the Ferret Model
Source: mBio. 2022 Aug 8;13(4):e01056-22. doi: 10.1128/mbio.01056-22 (PMC9426601; doi:10.1128/mbio.01056-22)
Supplement: TABLE S1 [file mbio.01056-22-s0004.pdf]

Supplementary Table S1. Summary of amino acid substitutions identified from whole genome sequencing in mixed infection ferrets treated with placebo, oseltamivir, baloxavir or a combination of oseltamivir and baloxavir

	Amino acid substitutions in A(H3N2) infected ferrets and number of ferrets in which the substitution was detected out of the total number in each antiviral treatment group			
Gene segment	Placebo	Oseltamivir	Baloxavir	Combination
PB2			S107G (1/3)	
PA	I38T (3/3)	I38T (3/3)	I38T (3/3)	I38T (3/3)<br>E677K (1/3)
NP	G384R (1/3)	S450N (1/3)		
HA	N181K (1/3)<br>T176K (1/3)	T327I (1/3)	N181K (1/3)	
NA			E259K (1/3)<br>L163Q (1/3)<br>E41G (1/3)	V240I(1/3)<br>D251H (1/3)
NS			N176I (1/3)	
	**Amino acid substitutions in A(H1N1pdm09) infected ferrets and number of ferrets in which the substitution was detected out of the total number in each antiviral treatment group**			
**Gene segment**	**Placebo**	**Oseltamivir**	**Baloxavir**	**Combination**
PA	E23K (3/3)	E23K (3/3)	E23K (3/3)	E23K (3/3)<br>S272R(1/3)<br>P325S (1/3)<br>R496Q (1/3)
NA		H275Y (3/3)		
NS				A257T (1/3)
