# Supplementary material for: Effect of Baloxavir and Oseltamivir in Combination on Infection with Influenza Viruses with PA/I38T or PA/E23K Substitutions in the Ferret Model
Source: mBio. 2022 Aug 8;13(4):e01056-22. doi: 10.1128/mbio.01056-22 (PMC9426601; doi:10.1128/mbio.01056-22)
Supplement: FIG S2 [file mbio.01056-22-s0002.pdf]

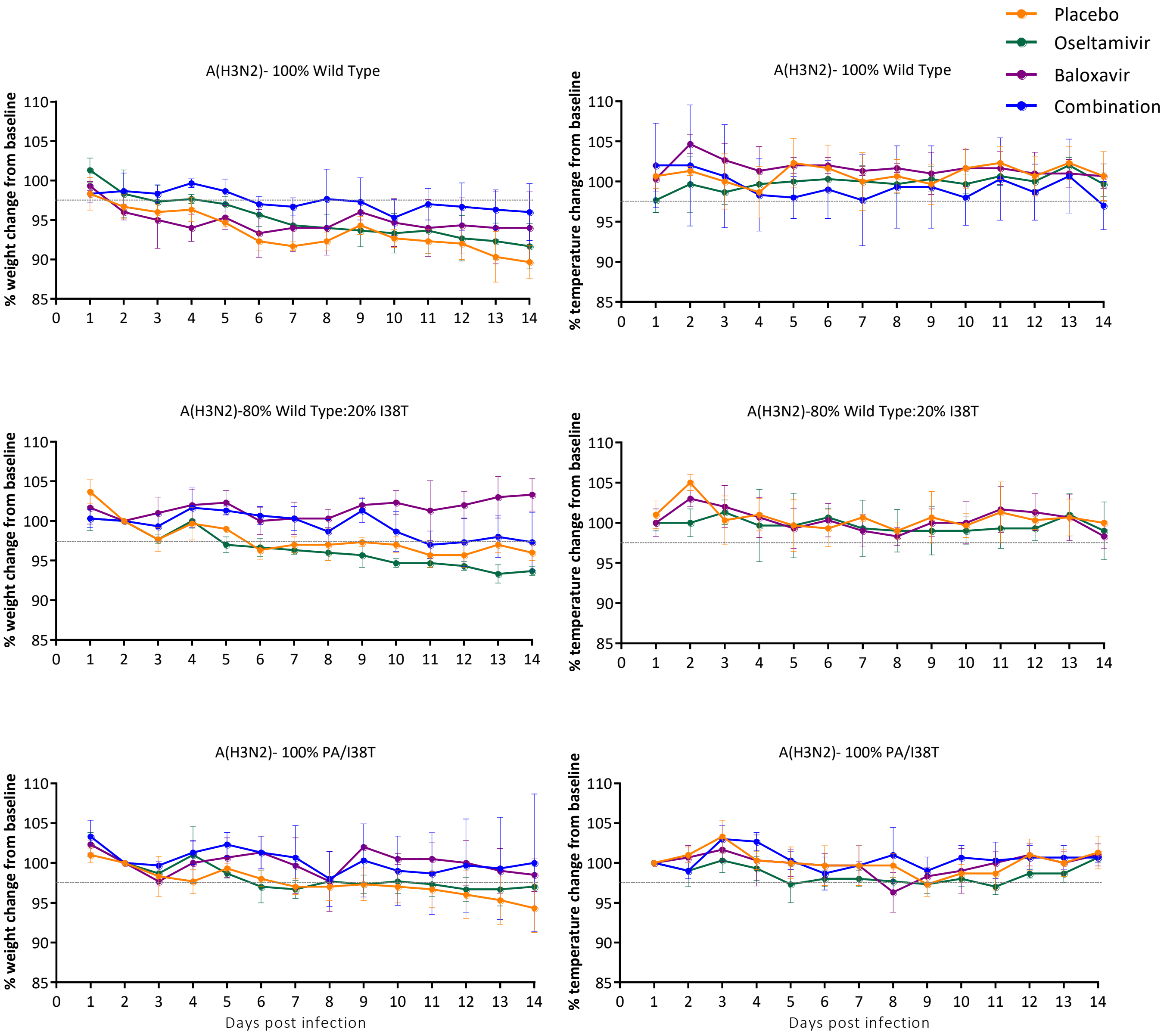

Supplementary Figure S2: Summary of percent change in weight and temperature of ferrets infected with the A(H3N2) clinical isolate pair compared to the starting baseline. Weight and temperature of ferrets were measured daily and the line plot depicts the mean and standard deviation in each ferret treatment group.
